# Bicyclic triterpenoid Iripallidal induces apoptosis and inhibits Akt/mTOR pathway in glioma cells

**DOI:** 10.1186/1471-2407-10-328

**Published:** 2010-06-24

**Authors:** Nitin Koul, Vivek Sharma, Deobrat Dixit, Sadashib Ghosh, Ellora Sen

**Affiliations:** 1 National Brain Research Centre, Manesar, Haryana 122 050, India

## Abstract

**Background:**

The highly resistant nature of glioblastoma multiforme (GBM) to chemotherapy prompted us to evaluate the efficacy of bicyclic triterpenoid Iripallidal against GBM in vitro.

**Methods:**

The effect of Iripallidal on proliferation and apoptosis in glioma cell lines was evaluated by MTS, colony formation and caspase-3 activity. The effect of iripallidal to regulate (i) Akt/mTOR and STAT3 signaling (ii) molecules associated with cell cycle and DNA damage was evaluated by Western blot analysis. The effect of Iripallidal on telomerase activity was also determined.

**Results:**

Iripallidal (i) induced apoptosis, (ii) inhibited Akt/mTOR and STAT3 signaling, (iii) altered molecules associated with cell cycle and DNA damage, (iv) inhibited telomerase activity and colony forming efficiency of glioma cells. In addition, Iripallidal displayed anti-proliferative activity against non-glioma cancer cell lines of diverse origin.

**Conclusion:**

The ability of Iripallidal to serve as a dual-inhibitor of Akt/mTOR and STAT3 signaling warrants further investigation into its role as a therapeutic strategy against GBM.

## Introduction

Iripallidal [(-) (6R,10S,11S,18R,22S)-26-Hydroxy-22-α-methylcycloirid-16-enal NSC 631939]- a bicyclic triterpenoid isolated from *Iris pallida *belongs to the terpenoid family as Paclitaxel. Paclitaxel is an effective chemotherapy for several types of neoplasms [1]. Iripallidal inhibited cell growth in a NCI 60 cell line screen [2] and induced cytotoxicity in human tumor cell lines [3]. Besides the fact that Iridals are ligands for phorbol ester receptors with modest selectivity for RasGRP3 [2], not much is known regarding its mechanism of action.

Despite recent advances in understanding molecular mechanisms involved in GBM progression, the prognosis of the most malignant brain tumor continues to be dismal. Ras activation occurs in GBMs [4] and this high level of active Ras has been a target for glioma therapy. RasGRP3- is an exchange factor that catalyzes the formation of the active GTP-bound form of Ras-like small GTPases [5]. Importantly, Ras activation stimulates its downstream effector Akt that plays a major role in glioblastoma development as ~80% of GBM cases express high Akt levels [6]. Akt activates mammalian target of rapamycin (mTOR), which is deregulated in glioblastoma [7]. mTOR phosphorylates p70 ribosomal S6 kinase (p70S6 kinase) that regulates translation of proteins involved in cellular proliferation and formation. Moreover, blocking mTOR signaling reduces glioma cell proliferation [8]. Given the importance of Akt/mTOR signaling in glioma cell survival, significant efforts are being invested in identifying inhibitors that target this pathway [8-10]. In addition to aberrant PI3K/Akt signaling; heightened STAT3 activation plays a critical role in glioblastoma and STAT3 inhibitors have shown promise as therapeutics for GBM [11-13]. In addition to RasGRP3 Iripallidal also binds to PKCα [2] which is known to induce cells ectopically expressing hyperactive Ras to undergo apoptosis [14]. Not only is STAT3 essential for Ras transformation [15] but constitutively activated STAT3 is negatively regulated by PKC-activated tyrosine phosphatase(s) [16]. As Iridals interacts with PKCα and RasGRP3-molecules that regulate Akt and STAT3 signaling, and since inhibition of Akt/mTOR and STAT3 signaling are being targeted for GBM treatment we evaluated the effect of Iripallidal on glioma cell proliferation and these signaling pathways.

## Materials and methods

### Cell culture and treatment

Glioblastoma cell lines A172, LN229, T98G and U87MG were obtained from American Type Culture Collection and cultured in DMEM supplemented with 10% fetal bovine serum. Peripheral blood mononuclear cells (PBMC) were isolated by Ficoll/Histopaque density gradient centrifugation. Adherent monocytes were purified from PBMC following adherence on glass petri-dish for three hours after flushing the non-adherent cells by extensive washing with PBS. All experiments with human PBMC were conducted under an approved institutional Human Ethics Committee protocol.

On attaining semi-confluence, cells were switched to serum free media and after 6 hours, cells were treated with different concentration of Iripallidal (in Dimethyl sulphoxide, DMSO) in serum free media for 24 hours. DMSO treated cells were used as controls. Iripallidal was purchased from Calbiochem, USA. All reagents were purchased from Sigma unless otherwise stated. Colon cancer cell line HT29, breast cancer line MCF-7, cervical cancer cell line HeLa, hepatocellular carcinoma cell line HepG2, acute myeloid leukemic cell line THP1 and human monocytes were similarly treated with Iripallidal.

### Determination of cell viability

Viability of Iripallidal treated monocytes and cancer cell lines was assessed using the [3-(4,5-dimethylthiazol-2-yl)-5-(3-carboxymethoxy-phenyl)-2-(4-sulfophenyl)- 2H-tetrazolium, inner salt] (MTS) (Promega) as described earlier [17].

### Assay of Caspase 3 activity

The Colorimetric Assay kits for caspase 3 (Sigma) were used to determine its enzymatic activity in Iripallidal treated glioma cells as described previously [18].

### Western Blot Analysis

Protein from whole cell lysates were isolated as described previously [19]. Protein (20-50 μg) isolated from control and Iripallidal treated cells was electrophoresed on 6% to 10% polyacrylamide gel and Western blotting performed as described [19]. Antibodies were purchased from Cell Signaling Technology (Danvers, MA) unless otherwise mentioned. The following antibodies were used: p21 (BD Biosciences), p27 (Abcam), pSTAT3 (Tyr705), pmTOR (Ser2448), mTOR, Akt, pAkt (Ser473), Cyclin D1 (Abcam), phospho-p70^S6K ^(Thr389), cMyc (Santa Cruz), phospho-S6K (Ser235/236), pH2AX Ser139 (Upstate), cleaved-PARP and β actin. Secondary antibodies were purchased from Vector Laboratories. After addition of chemiluminescence reagent (Amersham), blots were exposed to Chemigenius, Bioimaging System (Syngene, UK) for developing and images were captured using Genesnap software (Syngene). The blots were stripped and reprobed with anti-β-actin to determine equivalent loading as described [19].

### *Te*lo*TAGGG *Telomerase PCR ELISA PLUS

Telomerase activity was determined using the *T*elo*TAGGG *Telomerase PCR ELISA PLUS kit (Roche, Germany) as described previously [18].

### Colony formation in soft agar

The soft agar colony formation assay was performed using CytoSelect™ 96-Well Cell Transformation Assay kit (Cell Biolabs, Inc), as described previously [20].

### Statistical Analysis

All comparisons between groups were performed using two-tailed Paired student's t-Test. All values of p less than 0.05 were taken as significant.

## Results

### Iripallidal decreases viability and induces apoptosis in glioma cells

**Figure 1:**
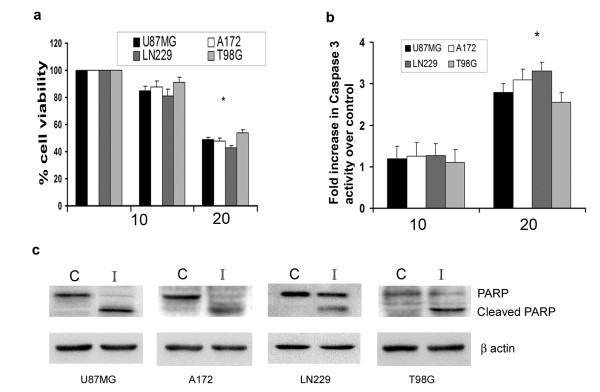
**Iripallidal decreases viability and induces apoptosis of glioma cells**. (a) Viability of Iripallidal treated glioma cells was determined by MTS assay. The graph represents the percentage viable cells of control observed when A172, LN229, T98G and U87MG cells were treated with 10 and 20μM concentration of Iripallidal for 24 hours. * Significant decrease from control (P < 0.05). (b) Increase in caspase-3 activity in Iripallidal treated A172, LN229, T98G and U87MG cells as determined by the Caspase-3 activity. * Significant increase from control (P < 0.05). (c) Treatment with Iripallidal increases cleaved PARP expression in glioma cells. Western blot analysis was performed to determine the expression of cleaved and native PARP in Iripallidal treated glioma cells. A representative blot is shown from three independent experiments with identical results. Blots were reprobed for β actin to establish equivalent loading. C and I denote Control and Iripallidal, respectively.

To determine whether Iripallidal affects viability of glioma cells, MTS assay was performed on A172, LN229, T98G and U87MG glioma cells treated with different concentrations of Iripallidal for 24 hours. While no significant cell death was observed in cells treated with 10 μM Iripallidal, a ~50% decrease in cell viability was observed in all the glioma cell lines tested upon treatment with 20 μM Iripallidal (Fig. 1a). Since the activation of caspase-3-like proteases is crucial in apoptotic cell death [21], we determined the caspase-3 activity in Iripallidal treated glioma cells. Decrease in viability was accompanied by a significant ~2.5 to 3-fold increase in caspase-3 activity in all the cell lines, as compared to control (Fig. 1b). As Caspase-3 activity was elevated in Iripallidal treated cells, we determined the expression of PARP in these cells. Treatment with Iripallidal elevated the level of cleaved PARP as compared to control, in all glioma cells tested (Fig. 1c). Increase in caspase-3 activation and cleaved-PARP level was indicative of apoptosis induction by Iripallidal. These results suggest that Iripallidal induce apoptosis in glioma cells.

### Iripallidal inhibits Akt/mTOR signaling in glioblastoma cells

**Figure 2:**
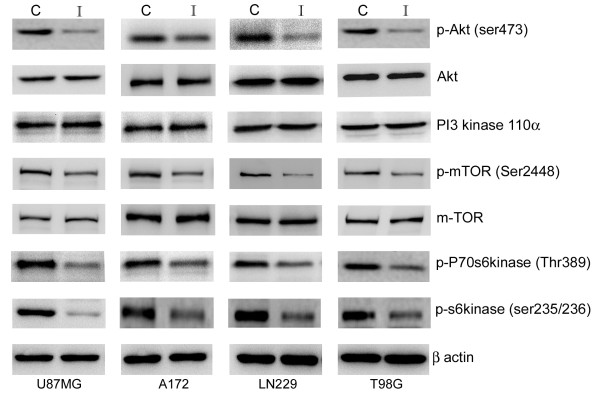
**Iripallidal inhibits Akt/mTOR signaling in glioma cells**. Western blot analysis was performed to determine the status of Akt/mTOR pathway in A172, LN229, T98G and U87MG glioma cells treated with 20 μM of Iripallidal for 24 hrs. A decrease in pAkt, pmTOR, pP70S6 kinase and pS6 kinase levels was observed upon Iripallidal treatment. Representative blot is shown from three independent experiments with identical results. C and I denote control and Iripallidal, respectively. Blots were reprobed for β-actin to establish equivalent loading.

As aberrant activation of the PI3K/Akt occurs frequently in glioblastomas [22], therapeutics approaches are directed towards targeting this pathway. Treatment with Iripallidal decreased Akt phosphorylation in glioma cells (Fig. 2). As inhibition of PI3 kinase p110α blocks Akt phosphorylation in glioma cells [23], we investigated whether this decrease in pAkt was the consequence of reduced p110α levels. Iripallidal had no effect on p110α levels (Fig. 2). As Iripallidal inhibited pAkt, we investigated its effect on Akt downstream target mTOR. Iripallidal downregulated phospho-mTOR in glioma cells (Fig. 2).

mTOR activation results in phosphorylation of effector molecule p70S6K and S6 ribosomal protein, which subsequently leads to mTOR-dependent gene transcription that regulates cell growth, protein synthesis, and metabolism. We therefore determined the effect of Iripallidal on the status of p70S6K and pS6 kinase. Iripallidal inhibited phosphorylation of mTOR targets 70S6K and ribosomal protein S6 (Fig. 2). These results indicate that iripallidal acts as a dual inhibitor of Akt/mTOR pathway.

### Iripallidal downregulates STAT3 phosphorylation in glioma cells

**Figure 3:**
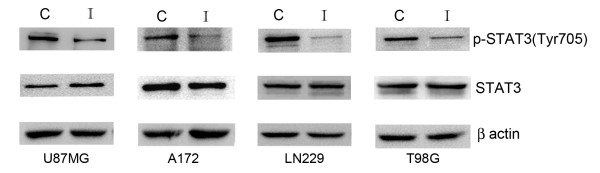
**Treatment with Iripallidal decreases STAT3 phosphorylation in glioma cells**. Western blotting analysis demonstrates a decrease in pSTAT3 levels in A172, LN229, T98G and U87MG glioma cells upon Iripallidal treatment. Representative blot is shown from three independent experiments with identical results. C and I denote control and Iripallidal, respectively. Blots were reprobed for β-actin to establish equivalent loading.

As mTOR inhibitor blocks STAT activation and glial differentiation [24] and since STAT3 inhibitors induce apoptosis in glioma cells [12], we determined the status of STAT3 activation in Iripallidal treated cells. A decrease in pSTAT3 Tyr705 was observed upon Iripallidal treatment (Fig. 3). These results indicate that Iripallidal inhibits STAT3 activation in glioma cells.

### Iripallidal affects expression of molecules involved in cell cycle regulation and DNA damage response

**Figure 4:**
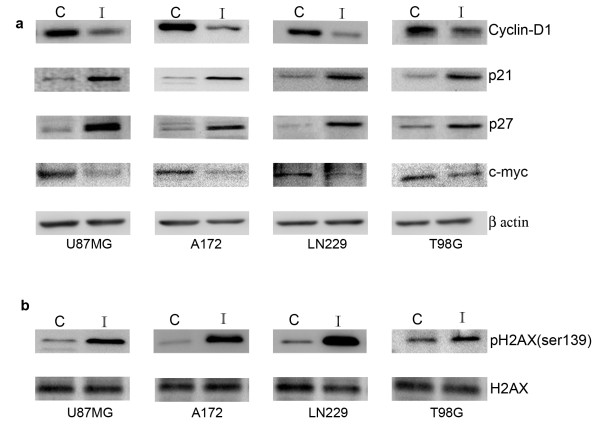
**Iripallidal effects expression of cell cycle regulators and DNA damage repair response molecules**. Representative blot is shown from three independent experiments with identical results. C and I denote control and Iripallidal, respectively. Western blot analysis reveals (a) increase in p21, p27 and decrease in cyclin D1, cMyc levels (b) increase in γH2AX in A172, LN229, T98G and U87MG glioma cells treated with 20 μM Iripallidal, as compared to control. Figures (a) and (b) are representative blot shown from three independent experiments with identical results. Blots were reprobed for β-actin to establish equivalent loading.

Inhibition of PI3-K/Akt/mTOR signaling effects cell cycle progression [23,25]. mTOR inhibitors induce cell cycle arrest through down regulation of Cyclin D and upregulation of p27 [8]. Since Iripallidal inhibited glioma cell proliferation, we determined the expression of molecules associated with cell cycle progression. An increase in p21 and p27, and decrease in cyclin D1 and cMyc levels was observed in glioma cells upon Iripallidal treatment (Fig. 4a).

As maintained DNA breaks induce apoptosis [26] and since H2AX is phosphorylated at sites of DNA double-strand breaks [27], we determined the expression of γ-H2AX in Iripallidal treated cells. While an increased γ-H2AX expression was observed in Iripallidal treated cells (Fig. 4b), the levels of total H2AX was unaffected (Fig. 4b).

### Iripallidal suppresses telomerase activity in glioma cells

**Figure 5:**
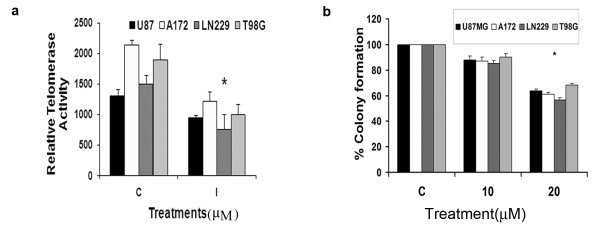
**Iripallidal decreases hTERT activity and colony forming ability of glioma cells**. (a) Glioma cells were treated with 20 μM Iripallidal and *T*elo*TAGGG *Telomerase PCR ELISA was performed. A decrease in hTERT activity was observed in Iripallidal treated cells as compared to control. (b) Iripallidal decreases the ability of glioma cells to form colonies in soft agar. Soft agar assay was performed on cells that were left untreated or treated with Iripallidal for 6 days. The graph indicates the percentage of colonies formed. Values in (a) and (b) represent the means ± SEM from 3 individual experiments. * Significant decrease from control (P < 0.05).

Inhibition of telomerase activity is an important anticancer modality since its inhibition causes apoptosis in human cancers [28]. Telomerase activity is regulated by Ras/PI3K/Akt pathway [29] and mTOR inhibitor rapamycin inhibits telomerase activity in endometrial cancer cells [30]. Besides, STAT3 regulates human telomerase reverse transcriptase (hTERT) expression in human cancer and primary cells [31]. Also, we have shown that inhibition of telomerase activity is associated with decrease glioma cell proliferation [18,20]. Since Iripallidal inhibits mTOR and STAT3 activation in glioma cells we investigated its ability to regulate telomerase activity. An approximate 50% reduction in telomerase activity was observed in glioma cells upon treatment with 20 μM Iripallidal (Fig. 5a). Telomerase inhibitors are known to reduce colony formation in soft agar assays [32] and STAT3 is essential for anchorage-independent growth of transformed cells [33]. Since Iripallidal decreased glioma cell survival we determined the ability of Iripallidal to effect the anchorage independent growth of glioma cells. Treatment with 20 μM Iripallidal reduced colony forming ability of glioma cells in soft agar by ~40%, as compared to control (Fig. 5b).

### Iripallidal inhibits proliferation of non-glioma cancer cells of diverse origin in vitro

**Figure 6:**
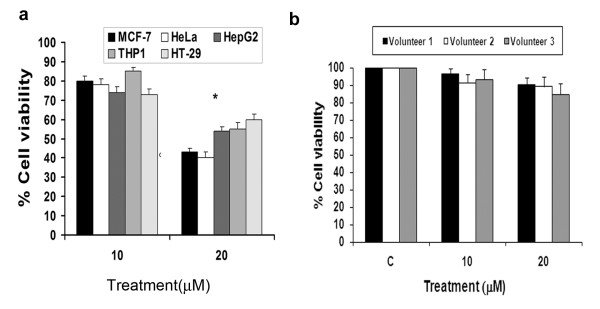
**Effect of Iripallidal on the viability of non-glioma cancer cell lines and normal human monocytes**. (a) Viability of Iripallidal treated non-glioma cancer cell lines and (b) normal human monocytes were determined by MTS assay. Graph represents the percentage viable MCF-7 (breast), HeLa (cervical), HepG2 (hepatocellular carcinoma), THP1 (acute myeloid leukemia), HT-29 (colon carcinoma) cells and monocytes treated with 10 and 20 μM concentration of Iripallidal for 24 hours, as determined by MTS assay. C denotes control. * Significant decrease from control (P < 0.05).

We next evaluated whether Iripallidal also exhibits anti-proliferative property against other human malignancies, by testing its effects against a panel of non-glioma human cancer cell lines in vitro. Treatment with 20 μM Iripallidal reduced viability of MCF-7, HeLa, HepG2, THP1 and HT-29 cells lines by ~35% to 60%, as compared to their respective controls (Fig. 6a). These findings indicate that Iripallidal not only inhibits proliferation of GBM, but also exhibits anti-proliferative activity against a wide variety of human cancers. To show the selectivity of Iripallidal for tumor cells, the effect of Iripallidal was investigated on normal human monocytes. Treatment of monocytes with Iripallidal induced ~8-10% decrease in viability, suggesting that the anti-proliferative ability of Iripallidal is selective for transformed cells (Fig. 6b).

## Discussion

In vitro screening of compounds with anticancer properties by NCI identified Iridals for their anti-proliferative activity. Besides its ability to bind PKCα and RasGRP3 [2], nothing is known regarding the mechanism of action or bioavailability of Iripallidal. Our studies suggest that Iripallidal induce apoptosis in glioma cells and inhibits the Akt/mTOR pathway. The efficacy of mTOR inhibitors in glioblastoma cell lines [8,10] has prompted their clinical trials for GBM [9,34]. As rapamycin activates Akt pathway by a negative feedback loop involving phosphorylation of insulin receptor substrate (IRS) by mTOR effector molecule S6 kinase [35,36], it was therefore not surprising that Rapamycin treatment induced Akt activation in some GBM patients in a Phase I clinical trial [9]. Moreover, dual inhibition of Akt and mTOR has proven effective in pre-clinical model of GBM [23], suggesting that dual Akt/mTOR inhibitor can effectively overcome the effects of feeback loop efficiently than a single inhibitor selectively targeting mTOR. As mTOR blockade is a biomarker of therapeutic efficacy in glioma [37], the unique ability of Iripallidal to inhibit both Akt and mTOR can be exploited as novel anti-glioma therapy. In addition to inhibiting Akt/mTOR axis, Iripallidal also inhibited STAT3 signaling. PKC inhibitor attenuates Ras activation and this attenuation correlates with an inhibition of RasGRP3 phosphorylation [38]. Interestingly, PKCα regulates mTOR [37] as well as STAT3 activation [16]. It is possible that Iripallidal effects Akt/mTOR and STAT3 signaling pathways through its ability to bind PKC.

Iripallidal mediated decrease in STAT3 activation was concurrent with decreased cyclin D1 and increased p21 expression. While cyclin D1 overexpression and STAT3 activation are mutually exclusive events [39], p21 inhibits STAT3 signaling [40]. Besides, inhibition of mTOR signaling induces cell cycle arrest through regulation of Cyclin D and p27 [8]. As telomerase inhibition is known to cause apoptosis in human cancers [28], the ability of Iripallidal to down-regulate telomerase activity may also represent a mechanism for its anti-proliferative effect on glioma cells. Besides glioma cell lines, Iripallidal also decreased the viability of several other cancer cell types although to different extents. It is known that cytotoxic responses is a reflection of an integrated readout of all targets and/or biochemical pathways affected upon drug exposure [41]. As strong co-relation exists between chemo-responsiveness and gene expression [41], it is likely that differential expression of cellular pathways in cancer cell types of diverse origin could have resulted in differences in sensitivity to Iripallidal.

Taken together our studies suggest that (i) Iripallidal induces glioma cell apoptosis and (ii) inhibits Akt/mTOR and STAT3 pathway. This ability of Iripallidal to act as a multi-inhibitor that blocks Akt/mTOR and STAT3 pathways suggest that its potential as a chemotherapeutic agent against GBM should be further evaluated. Importantly, Iripallidal is not only a promising candidate for the treatment of GBM but a wide variety of malignancies, since it elicits cell death in many tumor cell types.

## Conflict of Interest

"Bicyclic triterpenoid Iripallidal as a novel anti-glioma and anti-neoplastic therapy in vitro" has been filed for Indian patent (#2915/DEL/2008) and International Patent (PCT/IN09/000336) through Department of Biotechnology, Govt. of India.

## Authors' contributions

VS and ES designed the research; NK, VS, DD and SG performed the experiments, VS and ES analyzed the data; ES wrote the paper. All authors read and approved the final version of the manuscript.

## Pre-publication history

The pre-publication history for this paper can be accessed here:

http://www.biomedcentral.com/1471-2407/10/328/prepub

## References

[B1] Holmes FA Walters RS Theriault RL Forman AD Newton LK Raber MN Buzdar AU Frye DK Hortobagyi GN Phase II trial of taxol, an active drug in the treatment of metastatic breast cancer J Natl Cancer Inst 1991 83 24 1797 1805 1683908 10.1093/jnci/83.24.1797-a

[B2] Shao L Lewin NE Lorenzo PS Hu Z Enyedy IJ Garfield SH Stone JC Marner FJ Blumberg PM Wang S Iridals are a novel class of ligands for phorbol ester receptors with modest selectivity for the RasGRP receptor subfamily Journal of medicinal chemistry 2001 44 23 3872 3880 10.1021/jm010258f 11689073

[B3] Bonfils JP Pinguet F Culine S Sauvaire Y Cytotoxicity of iridals, triterpenoids from Iris, on human tumor cell lines A2780 and K562 Planta medica 2001 67 1 79 81 10.1055/s-2001-10625 11270729

[B4] Guha A Lau N Huvar I Gutmann D Provias J Pawson T Boss G Ras-GTP levels are elevated in human NF1 peripheral nerve tumors Oncogene 1996 12 3 507 513 8637706

[B5] Ebinu JO Bottorff DA Chan EY Stang SL Dunn RJ Stone JC RasGRP, a Ras guanyl nucleotide- releasing protein with calcium- and diacylglycerol-binding motifs Science (New York, NY) 1998 280 5366 1082 1086 10.1126/science.280.5366.1082 9582122

[B6] Sonoda Y Ozawa T Aldape KD Deen DF Berger MS Pieper RO Akt pathway activation converts anaplastic astrocytoma to glioblastoma multiforme in a human astrocyte model of glioma Cancer research 2001 61 18 6674 6678 11559533

[B7] Guertin DA Sabatini DM Defining the role of mTOR in cancer Cancer cell 2007 12 1 9 22 10.1016/j.ccr.2007.05.008 17613433

[B8] Paternot S Roger PP Combined inhibition of MEK and mammalian target of rapamycin abolishes phosphorylation of cyclin-dependent kinase 4 in glioblastoma cell lines and prevents their proliferation Cancer research 2009 69 11 4577 4581 10.1158/0008-5472.CAN-08-3260 19458076

[B9] Cloughesy TF Yoshimoto K Nghiemphu P Brown K Dang J Zhu S Hsueh T Chen Y Wang W Youngkin D Antitumor activity of rapamycin in a Phase I trial for patients with recurrent PTEN-deficient glioblastoma PLoS medicine 2008 5 1 e8 10.1371/journal.pmed.0050008 18215105 PMC2211560

[B10] Wei LH Su H Hildebrandt IJ Phelps ME Czernin J Weber WA Changes in tumor metabolism as readout for Mammalian target of rapamycin kinase inhibition by rapamycin in glioblastoma Clin Cancer Res 2008 14 11 3416 3426 10.1158/1078-0432.CCR-07-1824 18519772

[B11] Hussain SF Kong LY Jordan J Conrad C Madden T Fokt I Priebe W Heimberger AB A novel small molecule inhibitor of signal transducers and activators of transcription 3 reverses immune tolerance in malignant glioma patients Cancer research 2007 67 20 9630 9636 10.1158/0008-5472.CAN-07-1243 17942891

[B12] Iwamaru A Szymanski S Iwado E Aoki H Yokoyama T Fokt I Hess K Conrad C Madden T Sawaya R A novel inhibitor of the STAT3 pathway induces apoptosis in malignant glioma cells both in vitro and in vivo Oncogene 2007 26 17 2435 2444 10.1038/sj.onc.1210031 17043651

[B13] Lo HW Cao X Zhu H Ali-Osman F Constitutively Activated STAT3 Frequently Coexpresses with Epidermal Growth Factor Receptor in High-Grade Gliomas and Targeting STAT3 Sensitizes Them to Iressa and Alkylators Clin Cancer Res 2008 14 19 6042 6054 10.1158/1078-0432.CCR-07-4923 18829483 PMC2707832

[B14] Zhu T Tsuji T Chen C Roles of PKC isoforms in the induction of apoptosis elicited by aberrant Ras Oncogene 29 7 1050 1061 10.1038/onc.2009.344 19838205 PMC3517889

[B15] Gough DJ Corlett A Schlessinger K Wegrzyn J Larner AC Levy DE Mitochondrial STAT3 supports Ras-dependent oncogenic transformation Science (New York, NY) 2009 324 5935 1713 1716 10.1126/science.1171721 PMC2840701 19556508

[B16] Oka M Sumita N Sakaguchi M Iwasaki T Bito T Kageshita T Sato K Fukami Y Nishigori C 12-O-tetradecanoylphorbol-13-acetate inhibits melanoma growth by inactivation of STAT3 through protein kinase C-activated tyrosine phosphatase(s) The Journal of biological chemistry 2009 284 44 30416 30423 10.1074/jbc.M109.001073 19755418 PMC2781596

[B17] Sharma V Tewari R Sk UH Joseph C Sen E Ebselen sensitizes glioblastoma cells to Tumor Necrosis Factor (TNFalpha)-induced apoptosis through two distinct pathways involving NF-kappaB downregulation and Fas-mediated formation of death inducing signaling complex International journal of cancer 2008 123 9 2204 2212 10.1002/ijc.23771 18709644

[B18] Sharma V Koul N Joseph C Dixit D Ghosh S Sen E HDAC inhibitor Scriptaid induces glioma cell apoptosis through JNK activation and inhibits telomerase activity Journal of cellular and molecular medicine 2009 19583803 10.1111/j.1582-4934.2009.00844.x PMC3823006

[B19] Sharma V Joseph C Ghosh S Agarwal A Mishra MK Sen E Kaempferol induces apoptosis in glioblastoma cells through oxidative stress Molecular cancer therapeutics 2007 6 9 2544 2553 10.1158/1535-7163.MCT-06-0788 17876051

[B20] Dixit D Sharma V Ghosh S Koul N Mishra PK Sen E Manumycin inhibits STAT3, telomerase activity and growth of glioma cells by elevating intracellular reactive oxygen species generation Free radical biology & medicine 2009 10.1016/j.freeradbiomed.2009.04.031 19409983

[B21] Kumar S Lavin MF The ICE family of cysteine proteases as effectors of cell death Cell death and differentiation 1996 3 3 255 267 17180094

[B22] Choe G Horvath S Cloughesy TF Crosby K Seligson D Palotie A Inge L Smith BL Sawyers CL Mischel PS Analysis of the phosphatidylinositol 3'-kinase signaling pathway in glioblastoma patients in vivo Cancer research 2003 63 11 2742 2746 12782577

[B23] Fan QW Knight ZA Goldenberg DD Yu W Mostov KE Stokoe D Shokat KM Weiss WA A dual PI3 kinase/mTOR inhibitor reveals emergent efficacy in glioma Cancer cell 2006 9 5 341 349 10.1016/j.ccr.2006.03.029 16697955 PMC2925230

[B24] Rajan P Panchision DM Newell LF McKay RD BMPs signal alternately through a SMAD or FRAP-STAT pathway to regulate fate choice in CNS stem cells The Journal of cell biology 2003 161 5 911 921 10.1083/jcb.200211021 12796477 PMC2172962

[B25] Zhang C Yang N Yang CH Ding HS Luo C Zhang Y Wu MJ Zhang XW Shen X Jiang HL S9, a novel anticancer agent, exerts its anti-proliferative activity by interfering with both PI3K-Akt-mTOR signaling and microtubule cytoskeleton PloS one 2009 4 3 e4881 10.1371/journal.pone.0004881 19293927 PMC2654064

[B26] Zhou BB Elledge SJ The DNA damage response: putting checkpoints in perspective Nature 2000 408 6811 433 439 10.1038/35044005 11100718

[B27] Furuta T Takemura H Liao ZY Aune GJ Redon C Sedelnikova OA Pilch DR Rogakou EP Celeste A Chen HT Phosphorylation of histone H2AX and activation of Mre11, Rad50, and Nbs1 in response to replication-dependent DNA double-strand breaks induced by mammalian DNA topoisomerase I cleavage complexes The Journal of biological chemistry 2003 278 22 20303 20312 10.1074/jbc.M300198200 12660252

[B28] Zhang X Mar V Zhou W Harrington L Robinson MO Telomere shortening and apoptosis in telomerase-inhibited human tumor cells Genes & development 1999 13 18 2388 2399 10.1101/gad.13.18.2388 PMC317024 10500096

[B29] Ram R Uziel O Eldan O Fenig E Beery E Lichtenberg S Nordenberg Y Lahav M Ionizing radiation up-regulates telomerase activity in cancer cell lines by post-translational mechanism via ras/phosphatidylinositol 3-kinase/Akt pathway Clin Cancer Res 2009 15 3 914 923 10.1158/1078-0432.CCR-08-0792 19188162

[B30] Zhou C Gehrig PA Whang YE Boggess JF Rapamycin inhibits telomerase activity by decreasing the hTERT mRNA level in endometrial cancer cells Molecular cancer therapeutics 2003 2 8 789 795 12939469

[B31] Konnikova L Simeone MC Kruger MM Kotecki M Cochran BH Signal transducer and activator of transcription 3 (STAT3) regulates human telomerase reverse transcriptase (hTERT) expression in human cancer and primary cells Cancer research 2005 65 15 6516 6520 10.1158/0008-5472.CAN-05-0924 16061629

[B32] Dikmen ZG Gellert GC Jackson S Gryaznov S Tressler R Dogan P Wright WE Shay JW In vivo inhibition of lung cancer by GRN163L: a novel human telomerase inhibitor Cancer research 2005 65 17 7866 7873 16140956 10.1158/0008-5472.CAN-05-1215

[B33] Schlessinger K Levy DE Malignant transformation but not normal cell growth depends on signal transducer and activator of transcription 3 Cancer research 2005 65 13 5828 5834 10.1158/0008-5472.CAN-05-0317 15994959 PMC2100417

[B34] Doherty L Gigas DC Kesari S Drappatz J Kim R Zimmerman J Ostrowsky L Wen PY Pilot study of the combination of EGFR and mTOR inhibitors in recurrent malignant gliomas Neurology 2006 67 1 156 158 10.1212/01.wnl.0000223844.77636.29 16832099

[B35] O'Reilly KE Rojo F She QB Solit D Mills GB Smith D Lane H Hofmann F Hicklin DJ Ludwig DL mTOR inhibition induces upstream receptor tyrosine kinase signaling and activates Akt Cancer research 2006 66 3 1500 1508 10.1158/0008-5472.CAN-05-2925 16452206 PMC3193604

[B36] Tremblay F Marette A Amino acid and insulin signaling via the mTOR/p70 S6 kinase pathway. A negative feedback mechanism leading to insulin resistance in skeletal muscle cells The Journal of biological chemistry 2001 276 41 38052 38060 11498541 10.1074/jbc.M106703200

[B37] Fan QW Cheng CK Nicolaides TP Hackett CS Knight ZA Shokat KM Weiss WA A dual phosphoinositide-3-kinase alpha/mTOR inhibitor cooperates with blockade of epidermal growth factor receptor in PTEN-mutant glioma Cancer research 2007 67 17 7960 7965 10.1158/0008-5472.CAN-07-2154 17804702 PMC2597547

[B38] Teixeira C Stang SL Zheng Y Beswick NS Stone JC Integration of DAG signaling systems mediated by PKC-dependent phosphorylation of RasGRP3 Blood 2003 102 4 1414 1420 10.1182/blood-2002-11-3621 12730099

[B39] Quintanilla-Martinez L Kremer M Specht K Calzada-Wack J Nathrath M Schaich R Hofler H Fend F Analysis of signal transducer and activator of transcription 3 (Stat 3) pathway in multiple myeloma: Stat 3 activation and cyclin D1 dysregulation are mutually exclusive events The American journal of pathology 2003 162 5 1449 1461 12707028 10.1016/S0002-9440(10)64278-2 PMC1851203

[B40] Coqueret O Gascan H Functional interaction of STAT3 transcription factor with the cell cycle inhibitor p21WAF1/CIP1/SDI1 The Journal of biological chemistry 2000 275 25 18794 18800 10.1074/jbc.M001601200 10764767

[B41] Covell DG Wallqvist A Huang R Thanki N Rabow AA Lu XJ Linking tumor cell cytotoxicity to mechanism of drug action: an integrated analysis of gene expression, small-molecule screening and structural databases Proteins 2005 59 3 403 433 10.1002/prot.20392 15778971

